# Development and properties of functional yoghurt enriched with postbiotic produced by yoghurt cultures using cheese whey and skim milk

**DOI:** 10.3389/fmicb.2023.1276268

**Published:** 2023-09-25

**Authors:** Sepideh Sadighbathi, Per E. J. Saris, Saber Amiri, Amin Yousefvand

**Affiliations:** ^1^Department of Comparative Biomedicine and Food Science, University of Padua, Padua, Italy; ^2^Department of Microbiology, Faculty of Agriculture and Forestry, University of Helsinki, Helsinki, Finland; ^3^Department of Food Science and Technology, Faculty of Agriculture, Urmia University, Urmia, Iran

**Keywords:** postbiotics, yoghurt starters, cheese whey, skim milk, syneresis

## Abstract

This study aimed to examine the effects of supplementation of postbiotics derived from *Streptococcus thermophilus* (ST) and *Lactobacillus delbrueckii* subsp*. bulgaricus* (LB) in cheese whey (CW) and skim milk (SM) on antioxidant activity, viability of yoghurt starters, and quality parameters of low-fat yoghurt during 22 days of storage. The LB-CW (*L delbrueckii* ssp. *bulgaricus* postbiotic-containing cheese whey) sample exhibited the highest antioxidant activity, with 18.71% inhibition (*p* > 0.05). This sample also showed the highest water holding capacity (77.93%; *p* < 0.05) and a trend toward receiving the most favorable sensory attributes (*p* > 0.05) compared to the other samples. The LB-CW and LB-SM yoghurt samples exhibited significantly higher body and texture scores compared to the ST-SM-fortified yoghurt (*p* < 0.05). However, there was no significant difference in the overall acceptability of the LB-SM and ST-SM yoghurt samples across both starters (*p* > 0.05). Such findings highlight the potential of postbiotics as functional ingredients to enhance the nutritional and sensory aspects of yoghurt, further contributing to its appeal as a health-promoting product.

## 1. Introduction

The widespread popularity and high consumption of yoghurt make it an appealing choice for incorporating various value-added ingredients, such as probiotic bacteria, prebiotics, plant fibers, and extracts (Fazilah et al., 2018). Postbiotics are another potential supplement derived from beneficial microorganisms, particularly lactic acid bacteria (LAB), that can be generated in culture media, food, or the intestine. While a universally accepted definition is lacking (Aguilar-Toalá et al., 2021; Sabahi et al., 2022; Thorakkattu et al., 2022), postbiotic constituents encompass diverse intracellular and extracellular compounds. However, it is generally acknowledged that the removal of bacterial cells is a necessary step (Wegh et al., 2019; Moradi et al., 2021). The resulting postbiotic solution contains compounds that are safe to consume, and also feature specific chemical structures and a long shelf life, making it suitable for use in food products (Aguilar-Toalá et al., 2018). Postbiotics are gaining interest due to their inherent stability during processing and storage, making them more suitable for regions lacking reliable cold chains. Unlike probiotics, which often experience die-off during storage, postbiotics maintain stability over time. Probiotic manufacturers use overages to ensure labeled viable cell counts, and the live-to-dead ratio can change, impacting efficacy. Unlike probiotics, postbiotics remain stable at room temperature for years, eliminating viability concerns and allowing fixed microorganism levels at production. This stability makes postbiotics a promising option for areas with storage challenges (Salminen et al., 2021).

Probiotic bacteria produce water-soluble bioactive compounds known as “Postbiotics,” which encompass various metabolites such as bioactive lipids like conjugated linoleic acid (CLA), antimicrobial peptides like bacteriocins (BACs), and exopolysaccharides (EPSs) (Aguilar-Toalá et al., 2018). These bioactive compounds offer a multitude of reported advantages, including anti-inflammatory, antimicrobial, anti-diabetic, anti-cancer, immunomodulatory, anti-atherosclerotic, and anti-obesity activities, as documented in recent literature (Dubey et al., 2012; Dahiya and Puniya, 2017; Aguilar-Toalá et al., 2018; Amiri et al., 2020, 2022). Bacterial EPSs, which are polysaccharide molecules, are secreted by certain bacteria into the culture media. EPSs have been extensively studied for their technological applications in the food industry due to their textural and rheological properties. Moreover, they have gained considerable attention recently for their functional properties. For example, emerging research has highlighted their immunomodulatory potential, anti-inflammatory, anti-biofilm, and antioxidant activities (Kumar et al., 2007; Amiri et al., 2021).

Functional foods have the potential to be enriched with postbiotics to enhance the host’s immune activity. A mouse model study demonstrated that the cell-free fraction of fermented milk effectively prevented *Salmonella* infection (Dunand et al., 2019). Presently, postbiotics derived from *Lactobacillus acidophilus* LA5 and *Bifidobacterium animalis* subsp. *lactis* BB-12 are being utilized in the production of functional foods, especially for cheese products (Sharafi et al., 2022), as well as for modified milk, with their effectiveness assessed in randomized clinical trials. For instance, *Bifidobacterium breve* and *S. thermophilus* postbiotics showed a reduction in the incidence of allergy-related symptoms in infants with a positive history of atopy during their early months of life; this effect persisted even after discontinuation of the preparation (Morisset et al., 2010). Additionally, these postbiotics were associated with a milder course of acute diarrhea in infants (Thibault et al., 2004). Notably, one of the active metabolites of *S. thermophilus* is the aforementioned 3′-GL (Perrin et al., 2000).

*S. thermophilus* and *L. delbrueckii* ssp. *bulgaricus* are commonly used bacteria in dairy product manufacturing. During the commercial production of probiotics, postbiotics are generated as byproducts and are often considered as waste. Instead of being discarded, the postbiotic solution waste product presents a cost-effective and biologically active alternative source to enhance the nutritional content and shelf life of yoghurt during storage. In many previous studies exploring the use of LAB postbiotics in food, researchers relied on de Man Rogosa and Sharpe (MRS) as a preparation medium for postbiotic solutions. However, there is particular importance in identifying new, inexpensive, and underutilized agro-industrial waste for postbiotic preparation. In this study, postbiotic solutions derived from *Streptococcus thermophilus* and *Lactobacillus delbrueckii* subsp*. bulgaricus* were prepared using cheese whey and skim milk, two innovative growth model media. These postbiotic solutions were subsequently incorporated into yoghurt in the form of powdered nutritional supplements, adding a functional dimension to the yoghurt. The effect of each postbiotic powder on the microbial, chemical, and sensory characteristics of the yoghurt that was enriched with the postbiotic formulations was investigated.

## 2. Materials and methods

### 2.1. Microorganisms and inoculums

Freeze-dried cultures of *Streptococcus thermophilus* and *Lactobacillus delbrueckii* subsp*. bulgaricus* (Chr. Hansen, DK-2970 Hørsholm, Denmark) were obtained and individually weighted as recommended by the manufacturer, and grown for 24 h at 37°C in M17 (Neogen, Michigan, United States) and de Man, Rogosa, Sharpe broth (MRS) (Neogen, Michigan, United States), respectively. The cultures were then maintained at 4°C and sub-cultured three times in the same medium before each experiment.

### 2.2. Preparation of postbiotics solutions

Before the postbiotic preparation, *S. thermophilus* and *L. delbrueckii* ssp. *bulgaricus* were cultured at 37°C for 24 h in M17 and MRS, respectively. Following incubation, 50 μL of bacteria culture was separately sub-cultured in plastic tubes containing 50 mL of media, which were incubated at 37°C overnight. Next, the bacteria culture biomass was harvested by centrifugation at 4000× *g* for 10 min at 20°C and washed twice with sterilized standard saline solution. Finally, the harvested cells were resuspended in 10 mL of ultra-high temperature (UHT) milk and used as a bacteria culture to use in the next step. Skim milk (SM) and cheese whey (CW), obtained from Best way, Haulerwijk, Netherlands, were used as cultures media for postbiotic preparation. They were prepared as follows: initially, the pH was adjusted to 4.5 with 5 N hydrochloric acid (Merck, Darmstadt, Germany), then autoclaved at 121°C for 15 min, and the precipitates were separated by centrifugation at 2360× *g* for 5 min. The pH of the media (50 mL) was adjusted to 4.5 and autoclaved at 121°C for 15 min in 100 mL flasks. To optimize the incubation temperature and time of production of the highest postbiotic concentration (Experimental design not included), the method of Amiri et al. (2020) and Amiri et al. (2021) was used for postbiotic preparation in cheese whey and skim milk, with some modifications. Briefly, four different fermentation batches were prepared: ST-SM (*S. thermophilus* postbiotic-containing skim milk solution), ST-CW (*S. thermophilus* postbiotic-containing cheese whey solution), LB-SM (*L. delbrueckii* ssp. *bulgaricus* postbiotic-containing skim milk solution), and LB-CW (*L. delbrueckii* ssp. *bulgaricus* postbiotic-containing cheese whey solution). The resulting batches of ST-SM, ST-CW, LB-SM, and LB-CW were incubated at 40°C for 68 h, 39.6°C for 68 h, 46°C for 64 h, and 42.1°C for 68 h, respectively. During this time, the advancement of bacterial growth was monitored through the assessment of solution pH, total titratable acidity (TTA), and the turbidity of the solutions visually at 12-h intervals. After production, all fermented batches were freeze-dried (Martin Christ, Osterode am Harz, Germany) at −60°C with 0.0046 mBar of pressure for 48 h (freeze-drying time). After completing the procedure, these freeze-dried powders of postbiotics produced by bacteria were stored in closed plastic containers in a freezer at −20°C.

### 2.3. Preparation of postbiotic yoghurts

**Figure 1:**
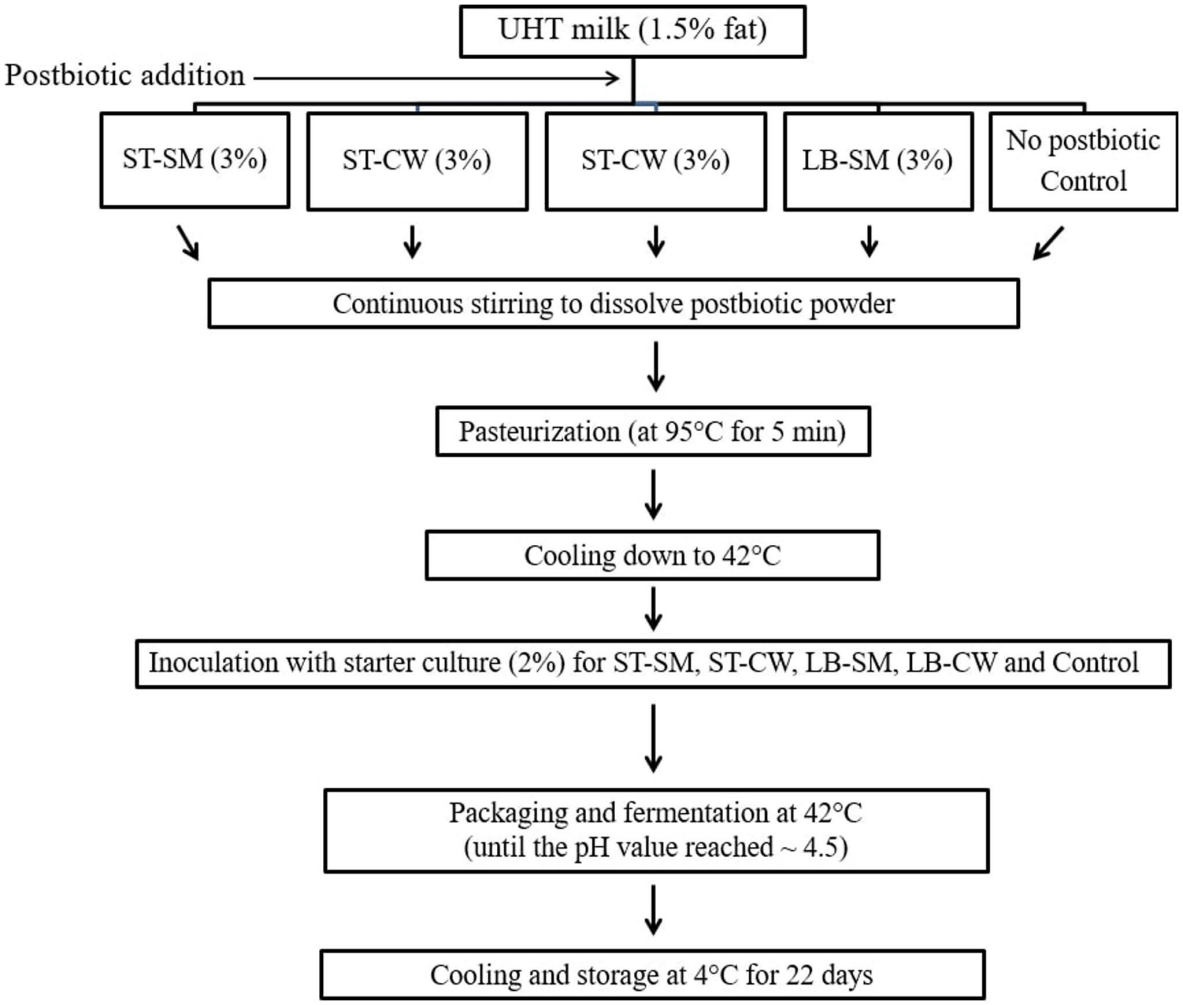
Low-fat yoghurt manufacturing flowchart. Control: yoghurt without postbiotic powder; ST-SM: yoghurt containing 3% *S. thermophilus* postbiotic-containing skim milk; ST-CW: yoghurt containing 3% *S. thermophilus* postbiotic-containing cheese whey; LB-SM: yoghurt containing 3% *L. delbrueckii* ssp. *bulgaricus* postbiotic-containing skim milk; LB-CW: yoghurt containing 3% *L. delbrueckii* ssp. *bulgaricus* postbiotic-containing cheese whey.

Low-fat yoghurt was manufactured according to the method of Ghaderi-Ghahfarokhi et al. (2020a) with some modifications. Commercial UHT milk (1.5 g/100 g of fat, 12.8 g/100 g of total solids (TS) content, and pH of 6.67) was used. Five yoghurt formulations, including Control (without postbiotic powder), ST-SM (yoghurt containing 3% *S. thermophilus* postbiotic-containing skim milk powder), ST-CW (yoghurt containing 3% *S. thermophilus* postbiotic-containing cheese whey powder), LB-SM (yoghurt containing 3% *L. delbrueckii* ssp. *bulgaricus* postbiotic-containing skim milk powder), and ST-CW (yoghurt containing 3% *L. delbrueckii* ssp. *bulgaricus* postbiotic-containing cheese whey powder) were prepared by the procedure depicted in Figure 1. The experimental batches were inoculated with a yoghurt starter culture, comprising *S. thermophilus* and *L. delbrueckii* ssp. *bulgaricus* at a concentration of 2% v/v. Following uniform agitation, the resulting yoghurts were packed into 100 mL sterile cups and subsequently incubated at 42°C until they reached a final pH of 4.5. Subsequently, the yoghurt samples were cooled to 4°C and stored for a duration of 22 days. Yoghurt production was performed in triplicate. The analysis encompassed the evaluation of both physicochemical attributes and microbial viability at four specific time points during the storage period: namely, days 1, 8, 15, and 22.

### 2.4. Physicochemical analysis of yoghurts

pH indexes of the yoghurt were measured using a pH meter (Thermo Orion Model-420A′). In addition, titratable acidity (TTA) of yoghurt samples was measured by the AOAC official method and expressed as % lactic acid (AOAC, 2005).

The syneresis values of yoghurt samples were determined as recommended by Tamime et al. (1996). Briefly, 25 g of each yoghurt batch was weighted on a Whatman paper No. 42 (Whatman) placed on the top of a funnel. Syneresis is expressed as the amount of whey separated from the samples under the force of gravity at 4°C for 2 h of drainage into a flask of known weight divided by the initial yoghurt mass.

The water holding capacity (WHC) of yoghurt samples was determined according to the centrifugation method reported by Sahan et al. (2008). Briefly, each 5 g yoghurt sample was weighted in a falcon tube (*M_i_*) and centrifuged at 3556× g for 30 min at 10°C. The resulting supernatant was discarded, and the expelled precipitate was collected and weighed (*M_p_*). WHC was calculated using the equation:


$$\mathrm{WHC}\;\left(\%\right)=\left[1-\left(M_{p}∕M_{i}\right)\right]\times 100$$

where *M_i_* and *M_p_* were the initial weight of the sample and the final weight of the precipitate, respectively.

### 2.5. Enumeration of starter cultures

The viability of *L. delbrueckii* ssp. *bulgaricus* and *S. thermophilus* was determined in freshly made yoghurt samples during the storage period as previously described, and expressed as log colony-forming units (CFU) per gram of product (log CFU/g). The yoghurt cup was agitated, and 1 g of each sample was mixed with 9 mL of physiological saline solution using a vortex mixer. Diluted samples were then enumerated using the pour-plate technique. In the count of *L. delbrueckii* ssp. *bulgaricus* and *S. thermophilus*, MRS agar and M17 agar were used, respectively. Both bacteria were incubated at 37°C for 72 h under anaerobic (*L*. *delbrueckii* ssp. *bulgaricus*) and aerobic (*S. thermophilus*) conditions, following Batawy and Khalil (2018).

### 2.6. Antioxidant activity determination

#### 2.6.1. Yoghurt samples extraction

The extraction method of yoghurt samples was conducted as reported by Demirci et al. (2017). To extract the desired components, 5 g of yoghurt was mixed with an appropriate amount of diluted methanol (80:20, methanol: distilled water) in a ratio of 25 mL. The mixture was then homogenized using an ultra-turrax homogenizer and subsequently centrifuged at 7200 rpm for 10 min at 4°C. The resulting mixture was filtered using Whatman No. 1 filter paper, and the liquid portion obtained after filtration was stored at 4°C for subsequent analysis of antioxidant activity.

#### 2.6.2. DPPH free radical scavenging activity assay

The DPPH radical activity was assessed as described by Yu et al. (2022). Initially, a solution of DPPH (0.01183 g) was prepared by dissolving it in 100 mL of 95% ethanol. Subsequently, 20 mL of the yoghurt sample was thoroughly mixed with 20 mL of the DPPH solution, followed by centrifugation at 10,000× *g* for 10 min. 2 mL of the resulting supernatant were combined with 8 mL of DPPH solution, mixed well, and left undisturbed in darkness for a duration of 30 min. Finally, the absorbance of the mixture was measured at a wavelength of 517 nm, using a blank solution of 95% ethanol, and the results are presented as the percent of DPPH cleared according to the formula:

DPPH clearance rate (%) = (1 – *A*_sample_/*A*_empty_) × 100%.

#### 2.6.3. ABTS^+^ free radical scavenging activity assay

The ABTS radical scavenging activity was measured according to the method of Yu et al. (2022). ABTS (7 mM) stock solution was prepared by dissolving ABTS in 2.45 Mm potassium persulfate solution, and stored in the dark at room temperature for 12–16 h. A working solution of ABTS was then created by mixing the stock solution with anhydrous ethanol to achieve a specific absorbance. For the analysis, a small amount of the sample was mixed with the ABTS working solution, shaken, and the absorbance was measured after a short incubation period. The same procedure was followed for the yoghurt samples. The results are expressed in ABTS clearance (%) form according to the following formula:

ABTS clearance rate (%) = (1 – *A*_sample_/0.700) 100%.

### 2.7. Sensory analysis

The sensory properties of yoghurt samples, including their visual appearance, texture, flavor, and mouth sensation, were assessed by 15 semi-trained panelists (staff, students, and researchers at the University of Helsinki, Helsinki, Finland). Yoghurt samples were served to the evaluators in 100-ml transparent glass cups bearing 3-digit random codes. The 10-point hedonic scale ranging from 1 (dislike very much) to 10 (like very much) was used on day 11 of storage. Yoghurt containers were labeled and the participants were trained to rinse their mouths before starting and between tasting the samples.

### 2.8. Statistical analysis

All physicochemical analyses and microbial counts were conducted in triplicates. The data obtained for yoghurt’s physicochemical, microbial, and sensorial evaluation were analyzed with ANOVA using the General Linear Model procedure, reported as mean ± standard deviations. Tukey’s test was used to compare the means; significant differences were estimated based on a *p* ≤ 0.05. All statistical analyses were carried out using Minitab 16 program (Minitab Inc., State College, PA, United States).

## 3. Results and discussion

### 3.1. pH and TTA of yoghurts

The pH values of the yoghurt samples were measured after 1, 8, 15, and 22 days of storage at 4°C. Our results showed that the postbiotic powder types and storage time had a significant effect on the pH value of the produced yoghurts (*p* < 0.05). On day 1 of the storage period, the index of pH of all yoghurt samples ranged between 4.62 and 4.68 (Table 1). This index decreased throughout the storage period, as also reported in other studies (Karaca et al., 2019; Ghaderi-Ghahfarokhi et al., 2020a). pH values of ST-CW varied from 4.66 to 4.63, and from 4.64 to 4.63 in LB-CW throughout the duration of storage (Table 1). This phenomenon was associated with the occurrence of organic acids present in the postbiotics that were assimilated by the yoghurt. A plausible explanation for this alteration could be attributed to mass exchange. Yoghurt samples containing cheese whey powders (ST-CW and LB-CW) showed a slight decrease in pH compared to skim milk (ST-SM and LB-SM) and Control formulations. The observed effect can also be attributed to the presence of organic acids in the absorbed postbiotics within the yoghurt. These results align with the research conducted by Sharafi et al. (2022), where it was observed that samples containing postbiotics demonstrated a significant reduction in pH values in comparison to the control samples. In another study, treatment with postbiotic decreased the pH values of the breast fillet samples compared to the control samples (İncili et al., 2021). The post-acidification phenomenon of all yoghurt formulations was seen (Table 1), which is primarily contributed to the continuity of fermentation by starter culture strains throughout the duration of shelf-life (Basiri et al., 2018). This can be observed by the slight decrease of pH in Control samples without any supplementation. At the end of storage, ST-CW, ST-SM, LB-CW, and LB-SM yoghurts displayed a pH drop of ~0.03, 0.09, 0.01, and 0.02 units compared to the first day, respectively, while the Control declined ~0.08 units. These results are in agreement with Elsamani and Ahmed (2014), who reported that pH values of yoghurts produced with or without cheese whey and skim milk were fairly similar, without noticeable difference between them.

**Table 1:** pH and titratable acidity (TTA; as lactic acid %) of low-fat yoghurts during 22 days of storage at 4°C.

		Storage period (days)			
Yoghurt formulation^1^		1	8	15	22
pH	Control	4.68 ± 0.05^ab,A^	4.64 ± 0.01^ab,B^	4.63 ± 0.02^a,BC^	4.60 ± 0.03^ab,C^
	ST-CW	4.66 ± 0.06^ab,A^	4.65 ± 0.02^ab,AB^	4.63 ± 0.01^aa,AB^	4.63 ± 0.01^a,B^
	ST-SM	4.68 ± 0.01^a,A^	4.66 ± 0.01^a,AB^	4.63 ± 0.02^a,B^	4.59 ± 0.04^b,C^
	LB-CW	4.64 ± 0.01^bc,A^	4.64 ± 0.07^ab,A^	4.64 ± 0.00^a,A^	4.63 ± 0.02^ab,A^
	LB-SM	4.62 ± 0.00^c,A^	4.62 ± 0.05^b,A^	4.61 ± 0.01^a,A^	4.60 ± 0.01^a,A^
TTA	Control	0.96 ± 0.04^a,A^	0.9 ± 0.08^a,A^	1.00 ± 0.08^a,A^	1.20 ± 0.00^a,A^
	ST-CW	0.76 ± 0.04^a,A^	0.86 ± 0.04^a,A^	0.76 ± 0.04^a,A^	1.03 ± 0.04^a,A^
	ST-SM	0.83 ± 0.04^a,A^	0.93 ± 0.04^a,A^	0.96 ± 0.04^a,A^	0.96 ± 0.04^a,A^
	LB-CW	0.96 ± 0.04^a,A^	0.86 ± 0.09^a,A^	0.90 ± 0.08^a,A^	1.00 ± 0.08^a,A^
	LB-SM	0.96 ± 0.04^a,A^	0.90 ± 0.08^a,A^	1.03 ± 0.012^a,A^	1.06 ± 0.09^a,A^

^a^Values (average ± SD) in the same column with the same superscript letters are not significantly different (*p* > 0.05). ^A^Values (average ± SD) in the same row with the same superscript letters are not significantly different (*p* > 0.05) between the storage days of each yoghurt sample.

^1^Abbreviations of different yoghurt formulations: Control (yoghurt without postbiotic); ST-SM (S. thermophilus postbiotic-containing skim milk); ST-CW (S. thermophilus postbiotic-containing cheese whey); LB-CW (L. delbrueckii ssp. bulgaricus postbiotic-containing cheese whey); and LB-SM (L. delbrueckii ssp. bulgaricus postbiotic-containing skim milk) (*n* = 3).

The most common acid produced by probiotic bacteria is lactic acid (Ghaderi-Ghahfarokhi et al., 2020b). As seen in Table 1, all yoghurts showed an increase in TTA and a decrease in pH during storage. In our study, the TTA index was distinctly related to the type of media and the strains used to produce postbiotic solutions, giving a higher concentration of lactic acid in LB-CW, LB-SM, and Control formulations as compared to ST-CW and ST-SM (Table 1). On day 8 of storage, the highest concentration of lactic acid was observed in the ST-SM formulation, although this was non-significant compered to others (*p* > 0.05). However, there were some fluctuations in the TTA values of the yoghurt during the storage period, consistent with other studies. For example, Gonzaíez-Martí et al. (2002) also observed a small change in the acid content, with no significant difference in the lactic acid production among the yoghurt samples enriched with different types of cheese whey powder.

### 3.2. Syneresis and WHC

**Figure 2:**
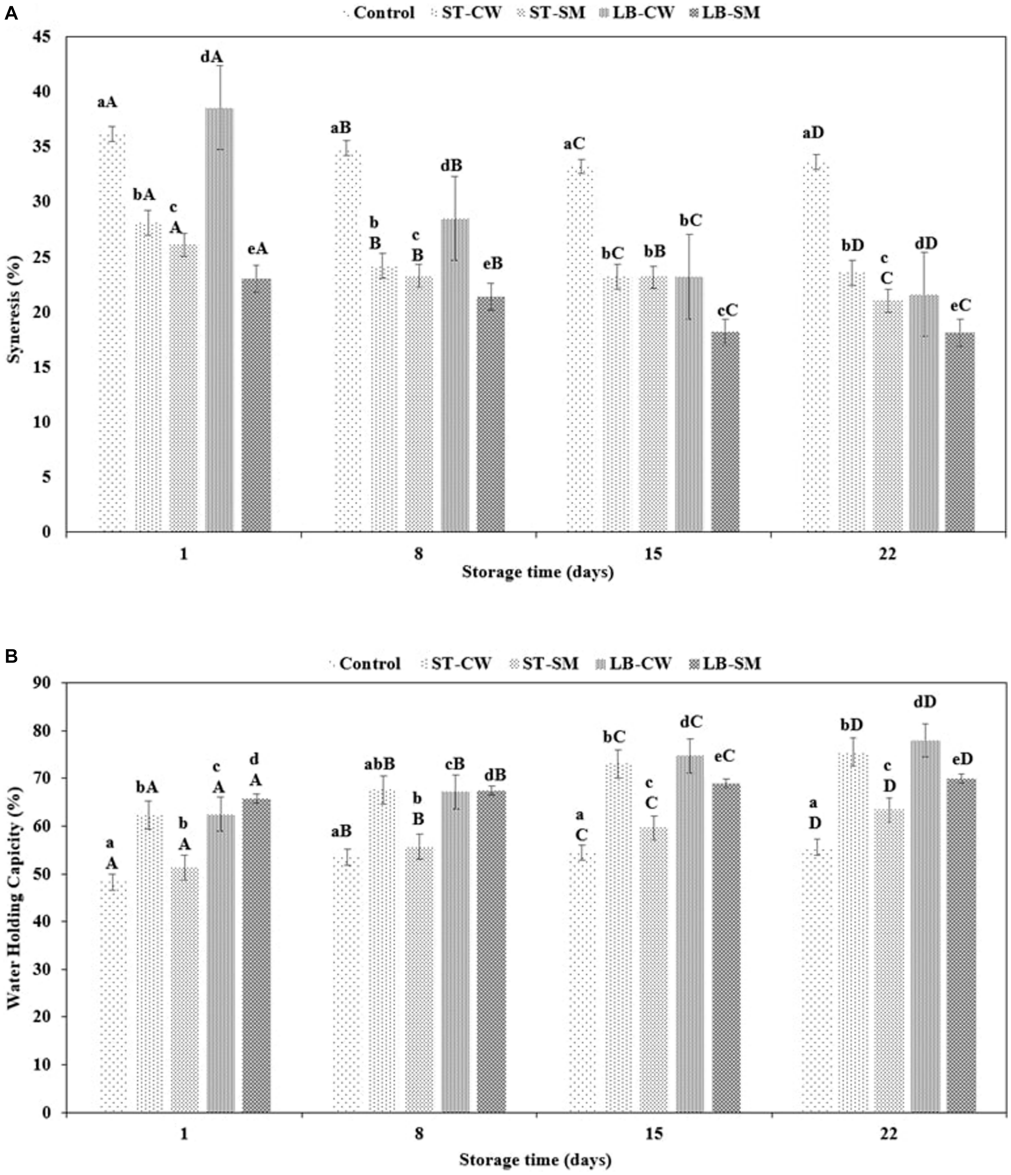
Syneresis (%) **(A)** and water holding capacity (%) **(B)** in different formulations of yoghurt during storage at 4°C. Control (yoghurt without postbiotic); ST-SM (*S. thermophilus* postbiotic-containing skim milk); ST-CW (*S. thermophilus* postbiotic-containing cheese whey); LB-CW (*L. delbrueckii* ssp. *bulgaricus* postbiotic-containing cheese whey); and LB-SM (*L. delbrueckii* ssp. *bulgaricus* postbiotic-containing skim milk). Lowercase letters indicate significant differences (*p* < 0.05) between the storage days of each yoghurt sample. Uppercase letters indicate significant differences (*p* < 0.05) between different samples at the same storage time. Error bars represent the mean (*n* = 3) ± standard deviation (SD).

Yoghurt’s coagulum stability is an important quality parameter that should be monitored during storage (Ghaderi-Ghahfarokhi et al., 2020b). As a result of weakening of the gel network, spontaneous syneresis causes the expulsion of whey from the body of yoghurt (Ozcan and Kurtuldu, 2014). The extent of syneresis was significantly influenced by yoghurt formulation and storage time (*p* < 0.05). Accordingly, the addition of CW or SM to reduce syneresis or improve yoghurt texture was noticeably dependent on the type of bacterial culture used. Whey separation varied across yoghurt samples within the range of 23.01–36.2% at the beginning of the experiment (*p* > 0.05). Throughout the cold storage period, all of the samples displayed a reduction in the rate of syneresis. Interestingly, the LB-CW yoghurt samples exhibited a significant decrease in syneresis from 38.58 to 21.60% (*p* < 0.05), while the control sample showed a minor decrease during storage. As depicted in Figure 2A, the LB-SM formulation exhibited the lowest syneresis rate among all samples, decreasing from 23.01% on day 1 of storage to 18.11% on day 22. A possible explanation can be the ability of the postbiotic compound, such as EPSs production by bacteria in LB-SM powder, to retain water in the yoghurt gel structure (Ghaderi-Ghahfarokhi et al., 2020a). In another research, Khider et al. (2022) demonstrated the impact of ESPs on diminishing and lessening syneresis in low-fat yogurt samples containing EPSs, as opposed to the control group. It is likely that the different conditions and strains used in the experiments have a major impact on the syneresis index (Gezginc et al., 2015). Our results accord with the findings of Akalin et al. (2012), who reported that casein-based samples showed firmer gels with less syneresis than yoghurts enriched with cheese whey.

The water holding capacity of a gel structure is an essential factor in yoghurt production, as it is an indicator of their ability to retain serum (whey) (Kpodo et al., 2014). Enriching yoghurt with CW and SM had a major impact on the WHC in yoghurt samples, with values ranging from 48.26 to 65.71% on Day 1 and Day 22 of storage, respectively (Figure 2B). Hence, CW and SM improve the tendency of yoghurts to retain water in comparison with Control samples. While the LB-SM formulation showed the most constant WHC (65.71–70.04%), the percentage of water retention was statistically decreased for other formulations (*p* < 0.05). The yoghurt samples enriched with cheese whey containing postbiotic powder of *S. thermophilus* (ST-CW) and *L. delbrueckii* ssp. *bulgaricus* (LB-CW) exhibited the highest WHC values of 77.93 and 75.47%, respectively. These findings are consistent with prior research conducted by Akalin et al. (2012) on yoghurt fortification using skim milk powder, whey protein concentration (WPC), and sodium calcium caseinate. The study reported a WHC index of 68.78% for yoghurt fortified with WPC during a 28-day storage period, indicating the highest water holding capacity among all formulations. Also, in line with our finding, a study by Delikanli and Ozcan (2014) stated that yoghurt samples enriched with CW exhibited the highest WHC (83.32%) compared to other formulations during a 14-day storage period. Another recent study revealed the impact of adding CW to yoghurt samples, noting a significant increase in WHC values during storage (Brodziak et al., 2020). As discussed in the previous paragraph, ESPs can also affect WHC of yoghurt. Khider et al. (2022) demonstrated the impact of EPSs on the water holding capacity of low-fat yoghurt that was fortified with varying concentrations of EPS derived from Leuconostoc strains, in comparison to a control sample. The study revealed a noticeable trend: as the concentration of EPS was elevated, there was a corresponding increase in the water holding capacity of the yoghurt.

### 3.3. Antioxidant activity

**Figure 3:**
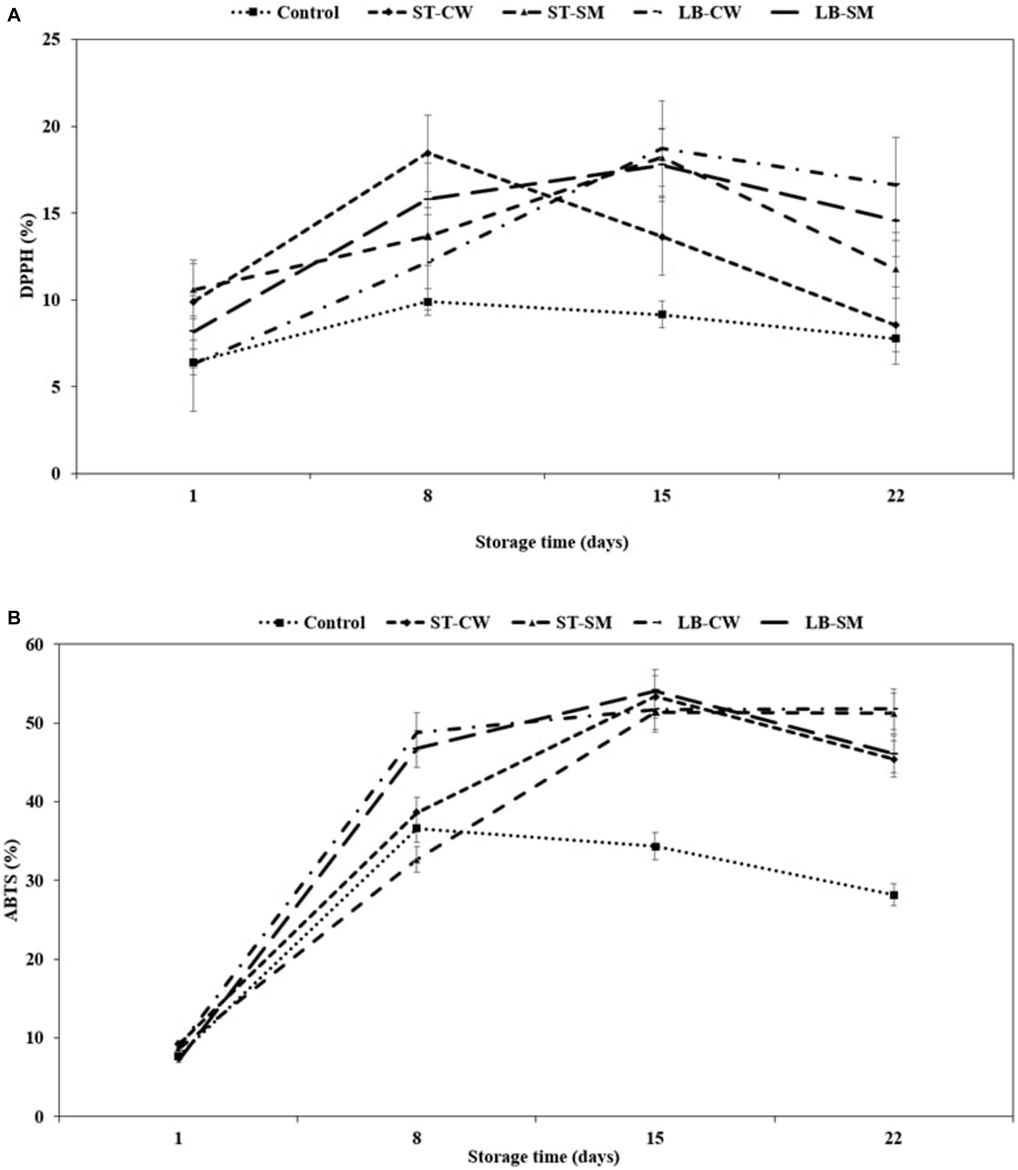
DPPH (%) **(A)** and ABTS (%) **(B)** in different formulations of yoghurt during storage at 4°C. Control (yoghurt without postbiotic); ST-SM (*S. thermophilus* postbiotic-containing skim milk); ST-CW (*S. thermophilus* postbiotic-containing cheese whey); LB-CW (*L. delbrueckii* ssp. *bulgaricus* postbiotic-containing cheese whey); and LB-SM (*L. delbrueckii* ssp. *bulgaricus* postbiotic-containing skim milk) (*n* = 3).

Postbiotics have been shown to possess a variety of functional/bioactive properties, including antioxidant activity, either directly (by interacting with the intestinal microbiota or immune cells) or indirectly (by interacting with other organs outside the gastrointestinal tract) (Sharma and Shukla, 2016; Aguilar-Toalá et al., 2018). EPSs and peptides are well-known postbiotic compounds with antioxidant properties. EPSs have been shown to reduce oxidative stress, lipid peroxidation, and inflammation. Peptides have been found to have anti-aging, anti-inflammatory, and anti-microbial effects. Peptides and EPSs both have potential applications in health-promoting foods and beverages (Sabeena Farvin et al., 2010; Amiri et al., 2019; Chang et al., 2021; Krunić and Rakin, 2022). In all yoghurt samples enriched with postbiotic supplement, the high rate of DPPH scavenging activity was significantly affected by yoghurt formulation and storage time (*p* < 0.05). As seen in Figure 3A, the LB-CW yoghurt sample showed the highest radical scavenging activity with 18.71% inhibition on day 15 of storage, which was significantly greater than all other yoghurt samples (*p* < 0.05) except ST-CW. The scavenging activities of DPPH radicals significantly increased with the addition of postbiotic powder compared to Control yoghurt. These findings are in agreement with Demirci et al. (2017), who reported that addition of rice bran, which has antioxidative properties, to yoghurt increased scavenging activities of DPPH radical (12.75%). Interestingly, DPPH activity of LB-CW was higher than the other samples on the last day of storage. In support of our findings, several previous studies have reported CW-enriched yoghurts can increase antioxidant activity (Bierzuńska and Cais-Sokolińska, 2018; Zoidou et al., 2019; Krunić and Rakin, 2022). However, Roumanas et al. (2016) stated that addition of cheese whey did not increase DPPH levels during storage.

In addition to the DPPH method, the ABTS method was also used to quantify the radical scavenging value to support quantified antioxidant activity. The initial ABTS activity ranged from 7.7 to 9.21% on the first day of storage, and it exhibited an exponential increase throughout the storage period, eventually reaching a relatively stable state after day 15 (Figure 3B). Yoghurts fortified with LB-CW and ST-SM showed higher ABTS activity on the final day of storage, with 51.78 and 51.19%, respectively (*p* < 0.05). The LB-CW sample exhibited the highest antioxidant activity in both DPPH and ABTS assays. This may be attributed to the ABTS radical inhibition capacities of EPSs produced by *L. delbrueckii* ssp. *bulgaricus* in postbiotic solutions. Abedfar et al. (2018) and El-Newary et al. (2017) reported that the percentage of ABTS radical scavenging activity of EPS increased with a rise in the concentration of EPS.

### 3.4. Viability of yoghurt cultures during yoghurt storage

**Figure 4:**
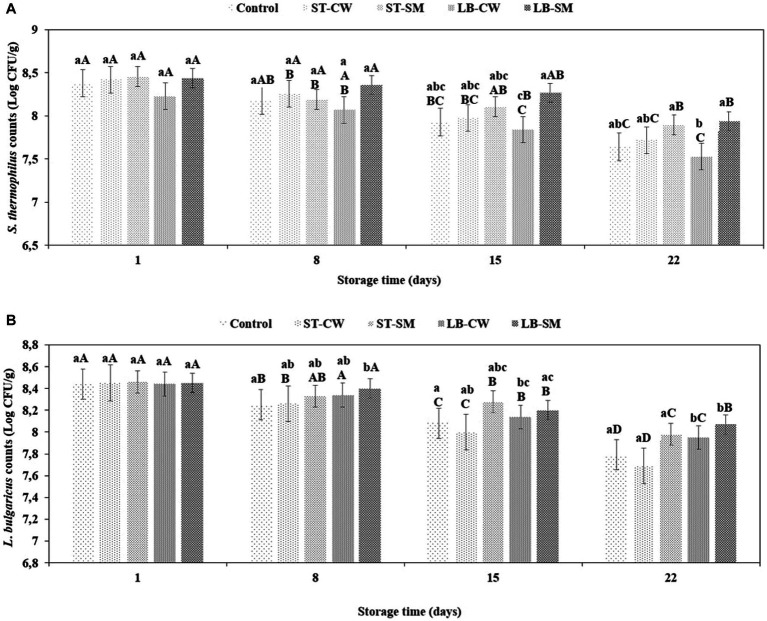
Viability of *S. thermophilus* **(A)** and *L. delbrueckii* ssp. *bulgaricus* **(B)** in different formulations of yoghurt during storage at 4°C. Control (yoghurt without postbiotic); ST-SM (*S. thermophilus* postbiotic-containing skim milk); ST-CW (*S. thermophilus* postbiotic-containing cheese whey); LB-CW (*L. delbrueckii* ssp. *bulgaricus* postbiotic-containing cheese whey); and LB-SM (*L. delbrueckii* ssp. *bulgaricus* postbiotic-containing skim milk). Lowercase letters indicate significant differences (*p* < 0.05) between the storage days of each yoghurt sample. Uppercase letters indicate significant differences (*p* < 0.05) between different samples at the same storage time. Error bars represent the mean (*n* = 3) ± standard deviation (SD).

As demonstrated in Figure 4, *S. thermophilus* and *L. delbrueckii* ssp. *bulgaricus* cell proportions were similar (approximately 10^8^ cfu/mL each) and maintained the same cell counts during the cold storage period. It is generally accepted that the standard count for *S. thermophilus* and *L. delbrueckii* ssp. *bulgaricus* should fluctuate around $10^{7}$ in yoghurt products (Fadela et al., 2009). In the current study, the viability of both yoghurt cultures was studied during a storage time of 22 days at 4°C. This cultures’ growth and survival were influenced by CW and SM addition during cold storage. After the first storage day, *S. thermophilus* and *L. delbrueckii* ssp. *bulgaricus* counts of ST-SM samples were 8.46 and 8.46 log cfu/g, respectively, which were higher than in other samples (*p* > 0.05) (Figures 4A,B). These results agree with the yoghurt culture counts reported in the literature: *S. thermophilus* counts in skim milk-fortified yoghurt increased to 9.78 log cfu/g on day 1 of storage (Marafon et al., 2011). During the first week of storage, the *S. thermophilus* and *L. delbrueckii* ssp. *bulgaricus* counts decreased slightly and continued to gradually decrease until the end of storage. Similarly, it was also found by Marafon et al., 2011 and Batawy and Khalil (2018) that the growth of yoghurt cultures decreased during cold storage. The viability of both starter cultures remained higher in the yoghurt fortified with LB-SM powder compared to the other samples during the storage period (*p* > 0.05) (Figures 4A,B). It is possible that LB-SM powder had more nutritional compounds that support yoghurt cultures. In the ST-CW and LB-CW yoghurt samples, the viable counts of starter cultures were 7.72 and 7.53 log cfu/g for *S. thermophilus* and 7.69 and 8.07 log cfu/g for *L. delbrueckii* ssp. *bulgaricus,* respectively, on day 22 of storage. These findings showed that the viable counts of starter cultures in the yoghurt samples were at favorable concentrations. It has been shown by Ranok et al. (2021) that adding cheese whey to yoghurt and increasing its concertation improves the bacteria viability in yoghurt products during storage and transit in the gastrointestinal tract. Furthermore, in a similar study by Glušac et al. (2015), the effects of adding honey and cheese whey to yoghurt were investigated, which revealed that adding cheese whey improved the viability of the yoghurt starters, but the addition of honey did not show a significant improvement.

### 3.5. Sensory analysis of yoghurts

The scores collected for sensory analyses (appearance, flavor, mouthfeel, body and texture, and overall acceptability) are displayed in Table 2. In the sensory analyses, the ST-SM samples received the lowest ratings in all indices except flavor by evaluators, while the highest ratings were given for the LB-CW and LB-SM yoghurt samples. This can be attributed to the development of texture and a more pleasant taste as a result of the postbiotic characteristics. The desirable body and texture in yoghurt samples (Table 2) could be associated with higher amounts of exopolysaccharide in the postbiotics powders (Aziznia et al., 2008; Amiri et al., 2019; Yousefvand et al., 2022). Our findings were in line with the studies reported by Salih and Hamid (2013) and Antunes et al. (2005) who showed addition of skim milk in the products has a positive impact on the flavor and viscosity of the samples. In terms of flavor, texture, mouthfeel, and overall acceptability, LB-CW-fortified yoghurt showed the highest scores (*p* > 0.05). Nevertheless, Akalin et al. (2012) reported no significant differences between experimental yoghurts containing and excluding CW in terms of sensory attributes. In order to determine overall acceptability, different sensory attributes must be considered, including flavor, texture, and appearance perceptions. In a related context, Ozma et al. (2022) unveiled that the application of an 8% solution of postbiotic derived from *Lactobacillus paracasei* ATCC 55544 as a coating for lamb meat slices resulted in consistent color, appearance, and overall consumer satisfaction ratings over the duration of storage. There were no notable alterations observed in these attributes for the lamb meat slices coated with the postbiotic. ST-SM and Control formulations were disliked slightly by panelists, while LB-CW and LB-SM formulations were preferred significantly by them. Antunes et al. (2005) found that the addition of WP and SM supplements had a positive impact on overall impressions.

**Table 2:** Sensory scores of low-fat yoghurts on day 11 of storage at 4°C.

Sensory attributes					
Yoghurt formulation^1^	Appearance	Flavor	Mouthfeel	Body and texture	Overall acceptability
Control	7.20 ± 2.01^ab^	6.33 ± 1.89^a^	7.53 ± 2.08^a^	7.73 ± 1.70^a^	7.40 ± 1.66^ab^
ST-CW	7.53 ± 2.18^ab^	7.33 ± 1.88^a^	7.46 ± 1.92^a^	7.13 ± 2.36^ab^	7.53 ± 2.06^ab^
ST-SM	6.26 ± 1.48^b^	6.93 ± 1.69^a^	7.00 ± 1.26^a^	6.06 ± 1.56^b^	6.66 ± 1.49^b^
LB-CW	8.00 ± 1.48^ab^	8.46 ± 1.69^a^	8.40 ± 1.26^a^	8.60 ± 1.56^a^	8.73 ± 1.49^a^
LB-SM	8.46 ± 1.54^a^	7.80 ± 1.46^a^	8.26 ± 1.48^a^	8.06 ± 1.56^a^	8.33 ± 1.34^ab^

^a-b^Values (average ± SD) in the same column with the same superscript letter are not significantly different (*p* > 0.05).

^1^Abbreviations of different yoghurt formulations: control (yoghurt without postbiotic); ST-SM (*S. thermophilus* postbiotic-containing skim milk); ST-CW (*S. thermophilus* postbiotic-containing cheese whey); LB-CW (*L. delbrueckii* ssp. *bulgaricus* postbiotic-containing cheese whey); and LB-SM (*L. delbrueckii* ssp. *bulgaricus* postbiotic-containing skim milk).

## 4. Conclusion

Emphasizing the significance of utilizing affordable and easily accessible sources for postbiotic production, whey – a byproduct regularly generated in cheese plants – is often overlooked and discarded as waste within the food industry. However, recognizing its potential, whey can serve as a valuable resource for postbiotic preparation. This study explored the use of cheese whey and skim milk as alternative sources for postbiotic preparation. Specifically, postbiotics were derived from *S. thermophilus* and *L. delbrueckii* ssp. *bulgaricus* in cheese whey and skim milk. Subsequently, the impact of these postbiotic-enriched cheese whey and skim milk supplements on the quality of yoghurt was thoroughly investigated. Postbiotic-enriched yoghurt showed high levels of antioxidant activity during 21 days of storage at 4°C. In addition to this beneficial property, sensory analysis conducted after 11 days of storage revealed that postbiotic-enriched yoghurt from *L. delbrueckii* ssp. *bulgaricus* in cheese whey and in skim milk were rated as highly acceptable – scores nearly reached the maximum rating. Moreover, the remaining yoghurt products also achieved satisfactory sensorial acceptance. Drawing upon observations related to syneresis, water holding capacity, and sensory evaluations throughout a refrigerated storage period, our results suggests that the postbiotic-enriched formula has the potential for practical use as a product. The incorporation of postbiotic-enriched powder into yoghurt did not exert a significant impact on the overall properties of the yoghurt, supporting its feasibility for application in the final product. Postbiotic solutions obtained from probiotics in cheese whey and skim milk show promising potential as nutritious liquids. Nevertheless, exploring postbiotic preparation using alternative animal and plant-based sources, particularly waste or byproducts, warrants further investigation. It is crucial to emphasize that regulations and proper labeling guidelines for food products containing postbiotics are essential prerequisites to enable their commercial utilization in the food industry.

## Data availability statement

The original contributions presented in the study are included in the article/Supplementary material, further inquiries can be directed to the corresponding author.

## Author contributions

SS: Investigation, Validation, Visualization, Conceptualization, Methodology, Writing – review & editing. PS: Validation, Visualization, Writing – review & editing, Conceptualization, Funding acquisition, Project administration, Resources. SA: Conceptualization, Methodology, Project administration, Validation, Visualization, Writing – review & editing. AY: Conceptualization, Funding acquisition, Methodology, Project administration, Resources, Supervision, Validation, Visualization, Writing – review & editing, Data curation, Formal analysis, Investigation, Software, Writing – original draft.

## References

[ref1] Abedfar A. Hosseininezhad M. Sadeghi A. Raeisi M. Feizy J. (2018). Investigation on “spontaneous fermentation” and the productivity of microbial exopolysaccharides by *Lactobacillus plantarum* and *Pediococcus pentosaceus* isolated from wheat bran sourdough. Food Sci. Technol. 96, 686–693. doi: 10.1016/j.lwt.2018.05.071

[ref2] Aguilar-Toalá J. E. Arioli S. Behare P. Belzer C. Canani R. B. Chatel J. M.. (2021). Postbiotics – when simplification fails to clarify. Nat. Rev. Gastroenterol. Hepatol. 18, 825–826. doi: 10.1038/s41575-021-00521-6, PMID: 34556825

[ref3] Aguilar-Toalá J. E. Garcia-Varela R. Garcia H. S. Mata-Haro V. González-Córdova A. F. Vallejo-Cordoba B.. (2018). Postbiotics: an evolving term within the functional foods field. Trends Food Sci. 75, 105–114. doi: 10.1016/j.tifs.2018.03.009

[ref4] Akalin A. S. Unal G. Dinkci N. Hayaloglu A. A. (2012). Microstructural, textural, and sensory characteristics of probiotic yogurts fortified with sodium calcium caseinate or whey protein concentrate. J. Dairy Sci. 95, 3617–3628. doi: 10.3168/jds.2011-5297 22720919

[ref5] Amiri S. Mokarram R. R. Khiabani M. S. Bari M. R. Alizadeh M. (2021). Optimization of food-grade medium for co-production of bioactive substances by *Lactobacillus acidophilus* LA-5 for explaining pharmabiotic mechanisms of probiotic. J. Food Sci. Technol. 58, 1–12. doi: 10.1007/s13197-020-04894-5, PMID: 34538890 PMC8405832

[ref6] Amiri S. Mokarram R. R. Khiabani M. S. Bari M. R. Khaledabad M. A. (2020). In situ production of conjugated linoleic acid by *Bifidobacterium lactis* BB12 and *Lactobacillus acidophilus* LA5 in milk model medium. Food Sci. Technol. 153:109933:112449. doi: 10.1016/j.lwt.2020.109933

[ref7] AOAC - Association of Official Analytical Chemists (2005) Official methods of analysis of AOAC international (18th Edn.). Gaithersburg, MD: Association of Official Analytical Chemists International, 7.

[ref8] Amiri S. Mokarram R. R. Khiabani M. S. Rari M. R. Khaledabad M. A. (2019). Exopolysaccharides production by *Lactobacillus acidophilus* LA5 and *Bifidobacterium animalis* subsp. lactis BB12: optimization of fermentation variables and characterization of structure and bioactivities. Int. J. Biol. Macromol. 123, 752–765. doi: 10.1016/j.ijbiomac.2018.11.084, PMID: 30447370

[ref9] Amiri S. Mokarram R. R. Khiabani M. S. Bari M. R. Khaledabad M. A. (2022). Characterization of antimicrobial peptides produced by *Lactobacillus acidophilus* LA-5 and *Bifidobacterium lactis* BB-12 and their inhibitory effect against foodborne pathogens. Food Sci. Technol. 153:112449. doi: 10.1016/j.lwt.2021.112449

[ref10] Antunes A. E. C. Cazetto T. F. Bolini H. M. A. (2005). Viability of probiotic micro-organisms during storage, postacidification and sensory analysis of fat-free yogurts with added whey protein concentrate. Int. J. Dairy Technol. 58, 169–173. doi: 10.1111/j.1471-0307.2005.00203.x

[ref11] Aziznia S. A. Khosrowshahi A. Madadlou A. Rahimi J. (2008). Whey protein concentrate and gum tragacanth as fat replacers in nonfat yogurt: chemical, physical, and microstructural properties. J. Dairy Sci. 91, 2545–2552. doi: 10.3168/jds.2007-0875, PMID: 18565911

[ref12] Batawy O. E. Khalil O. S. (2018). Production and properties of low-fat set yoghurt made with Jerusalem artichoke powder. J Prob Health. 6, 77–90. doi: 10.4172/2329-8901.1000192

[ref13] Bierzuńska P. Cais-Sokolińska D. (2018). Determination of antioxidant activity of yoghurt enriched with polymerized whey protein. Mljekarstvo. 68, 272–281. doi: 10.15567/mljekarstvo.2018.040

[ref14] Brodziak A. Król J. Barłowska J. Teter A. Florek M. (2020). Changes in the physicochemical parameters of yoghurts with added whey protein in relation to the starter bacteria strains and storage time. J. Anim. 10:1350. doi: 10.3390/ani10081350, PMID: 32759770 PMC7460345

[ref15] Basiri S. Haidary N. Shekarforoush S. S. Niakousari M. (2018). Flaxseed mucilage: a natural stabilizer in stirred yogurt. Carbohydr. Polym. 1, 59–65. doi: 10.1016/j.carbpol.2018.01.049 29486845

[ref16] Chang H. M. Foo H. L. Loh T. C. Lim E. T. C. Abdul Mutalib N. E. (2021). Comparative studies of inhibitory and antioxidant activities, and organic acids compositions of postbiotics produced by probiotic Lactiplantibacillus plantarum strains isolated from Malaysian foods. Front. Vet. Sci. 7:602280. doi: 10.3389/fvets.2020.60228, PMID: 33575277 PMC7870707

[ref17] Dahiya D. K. Puniya A. K. (2017). Isolation, molecular characterization and screening of indigenous lactobacilli for their abilities to produce bioactive conjugated linoleic acid (CLA). J. Food Sci. Technol. 54, 792–801. doi: 10.1007/s13197-017-2523-x, PMID: 28298694 PMC5334239

[ref18] Delikanli B. Ozcan T. (2014). Effects of various whey proteins on the physicochemical and textural properties of set type nonfat yoghurt. Int. J. Dairy Technol. 67, 495–503. doi: 10.1111/1471-0307.12142

[ref19] Demirci T. Aktaş K. Sözeri D. Öztürk H. İ. Akın N. (2017). Rice bran improve probiotic viability in yoghurt and provide added antioxidative benefits. J. Funct. Foods 36, 396–403. doi: 10.1016/j.jff.2017.07.019

[ref20] Dubey V. Ghosh A. R. Mandal B. K. (2012). Appraisal of conjugated linoleic acid production by probiotic potential of Pediococcus spp. GS4. Appl. Biochem. Biotechnol. 168, 1265–1276. doi: 10.1007/s12010-012-9855-9, PMID: 22971829

[ref21] Dunand E. Burns P. Binetti A. Bergamini C. Peralta G. H. Forzani L.. (2019). Postbiotics produced at laboratory and industrial level as potential functional food ingredients with the capacity to protect mice against Salmonella infection. J. Appl. Microbiol. 127, 219–229. doi: 10.1111/jam.14276 30973185

[ref22] El-Newary S. A. Ibrahim A. Y. Asker M. S. Mahmoud M. G. El Awady M. E. (2017). Production, characterization and biological activities of acidic exopolysaccharide from marine *Bacillus amyloliquefaciens* 3MS 2017. Asian Pac J Trop Med 10, 652–662. doi: 10.1016/j.apjtm.2017.07.005 28870341

[ref23] Elsamani M. O. Ahmed I. A. M. (2014). Physicochemical characteristics and organoleptic properties of peanuts milk-based yoghurt fortified with skimmed milk powder. Res. J. Appl. Sci. 1, 68–72.

[ref24] Fadela C. Abderrahim C. Ahmed B. (2009). Sensory and physicochemical characteristic of yoghurt manufactured with ewes and skim milk. World J. dairy Food Sci. 4, 136–140.

[ref25] Fazilah N. F. Ariff A. B. Khayat M. E. Rios-Solis L. Halim M. (2018). Influence of probiotics, prebiotics, synbiotics and bioactive phytochemicals on the formulation of functional yogurt. J. Funct. Foods 48, 387–399. doi: 10.1016/j.jff.2018.07.039

[ref26] Gezginc Y. Topcal F. Comertpay S. Akyol I. (2015). Quantitative analysis of the lactic acid and acetaldehyde produced by Streptococcus thermophilus and *Lactobacillus bulgaricus* strains isolated from traditional Turkish yogurts using HPLC. J. Dairy Sci. 98, 1426–1434. doi: 10.3168/jds.2014-8447, PMID: 25547312

[ref27] Ghaderi-Ghahfarokhi M. Yousefvand A. Gavlighi H. A. Zarei M. Farhangnia P. (2020a). Developing novel synbiotic low-fat yoghurt with fucoxylogalacturonan from tragacanth gum: investigation of quality parameters and *Lactobacillus casei* survival. Food Sci. Nutr. 8, 4491–4504. doi: 10.1002/fsn3.1752, PMID: 32884729 PMC7455973

[ref28] Ghaderi-Ghahfarokhi M. Yousefvand A. Gavlighi H. A. Zarei M. (2020b). The effect of hydrolysed tragacanth gum and inulin on the probiotic viability and quality characteristics of low-fat yoghurt. Int. J. Dairy Technol. 74, 161–169. doi: 10.1111/1471-0307.12742

[ref29] Glušac J. Stijepić M. Đurđević-Milošević D. Milanović S. Kanurić K. Vukić V. (2015). Growth and viability of *Lactobacillus delbrueckii* subsp. bulgaricus and *Streptococcus thermophilus* in traditional yoghurt enriched by honey and whey protein concentrate. Iran. J. Vet. Res. 16, 249–254. PMID: 27175184 PMC4782693

[ref30] Gonzaíez-Martí C. G. Becerra M. Chafer M. Albors A. Carot J. M. Chiralt A. (2002). Influence of substituting milk powder for whey powder on yoghurt quality. Trends Food Sci. 13, 334–340. doi: 10.1016/S0924-2244(02)00160-7

[ref31] İncili G. K. Karatepe P. Akgöl M. Kaya B. Kanmaz H. Hayaloğlu A. A. (2021). Characterization of *Pediococcus acidilactici* postbiotic and impact of postbiotic-fortified chitosan coating on the microbial and chemical quality of chicken breast fillets. Int. J. Biol. Macromol. 184, 429–437. doi: 10.1016/j.ijbiomac.2021.06.106, PMID: 34166693

[ref32] Karaca O. B. Güzeler N. Tangüler H. Yasar K. Akın M. B. (2019). Effects of apricot fiber on the physicochemical characteristics, the sensory properties and bacterial viability of nonfat probiotic yoghurts. Foods 8:33. doi: 10.3390/foods8010033, PMID: 30669321 PMC6352206

[ref33] Khider M. El-Readi M. Z. Abdalrahim S. Zohri A. N. Ibrahim I. M. Abulreesh H. H. (2022). Functional low-fat set yogurt enhanced with microbial exo-polysaccharides-mediated anticancer activity. J Pure Appl Microbiol 16, 2601–2618. doi: 10.22207/JPAM.16.4.28

[ref34] Kpodo F. M. K. Afoakwa E. O. Amoa B. B. Budu A. S. Saalia F. K. (2014). Effect of ingredient variation on microbial acidification, susceptibility to syneresis, water holding capacity and viscosity of soy-peanut-cow milk yoghurt. J. Nutri. Health Food Eng. 1, 74–79. doi: 10.15406/jnhfe.2014.01.00012

[ref35] Krunić T. Rakin M. B. (2022). Enriching alginate matrix used for probiotic encapsulation with whey protein concentrate or its trypsin-derived hydrolysate: impact on antioxidant capacity and stability of fermented whey-based beverages. Food Chem. 370:130931. doi: 10.1016/j.foodchem.2021.130931 34509939

[ref36] Kumar A. S. Mody K. Jha B. (2007). Bacterial exopolysaccharides–a perception. J. Basic Microbiol. 47, 103–117. doi: 10.1002/jobm.200610203, PMID: 17440912

[ref37] Marafon A. P. Sumi A. Granato D. Alcântara M. R. Tamime A. Y. Nogueira de Oliveira M. (2011). Effects of partially replacing skimmed milk powder with dairy ingredients on rheology, sensory profiling, and microstructure of probiotic stirred-type yoghurt during cold storage. J. Dairy Sci. J. 94, 5330–5340. doi: 10.3168/jds.2011-4366, PMID: 22032355

[ref38] Moradi M. Molaei R. Guimarães J. T. (2021). A review on preparation and chemical analysis of postbiotics from lactic acid bacteria. Enzym. Microb. Technol. 143:109722. doi: 10.1016/j.enzmictec.2020.109722, PMID: 33375981

[ref39] Morisset M. Aubert-Jacquin C. Soulaines P. Moneret-Vautrin D. A. Dupont C. (2010). A non-hydrolyzed, fermented milk formula reduces digestive and respiratory events in infants at high risk of allergy. Eur. J. Clin. Nutr. 65, 175–183. doi: 10.1038/ejcn.2010.250, PMID: 21081959

[ref40] Ozcan T. Kurtuldu O. (2014). Influence of dietary fiber addition on the properties of probiotic yogurt. Int. J. Chem. Eng. 5, 397–401. doi: 10.7763/IJCEA.2014.V5.417

[ref41] Ozma M. A. Abbasi A. Sabahi S. (2022). Characterization of postbiotics derived from *Lactobacillus paracasei* ATCC 55544 and its application in *malva sylvestris* seed mucilage edible coating to the improvement of the microbiological, and sensory properties of lamb meat during storage. Biointerface Res. Appl. Chem. 13:267. doi: 10.33263/BRIAC133.267

[ref42] Perrin V. Fenet B. Praly J. P. Lecroix F. Ta C. D. (2000). Identification and synthesis of a trisaccharide produced from lactose by transgalactosylation. Carbohydr. Res. 325, 202–210. doi: 10.1016/S0008-6215(99)00309-2, PMID: 10795811

[ref43] Ranok A. Kupradit C. Khongla C. Musika S. Mangkalanan S. Suginta W. (2021). Effect of whey protein concentrate on probiotic viability and antioxidant properties of yoghurt during storage and simulated gastrointestinal transit. Int. Food Res. J. 28, 110–119. doi: 10.47836/ifrj.28.1.11

[ref44] Roumanas D. Moatsou G. Zoidou E. Sakkas L. Moschopoulou E. (2016). Effect of enrichment of bovine milk with whey proteins on biofunctional and rheological properties of low fat yoghurt-type products. Curr. Res. Nutr. Food Sci. 4, 105–113. doi: 10.12944/CRNFSJ.4

[ref45] Sabahi S. Homayouni Rad A. Aghebati-Maleki L. Sangtarash N. Ozma M. A. Karimi A.. (2022). Postbiotics as the new frontier in food and pharmaceutical research. Crit. Rev. Food Sci. Nutr. 29, 1–28. doi: 10.1080/10408398.2022.2056727 35348016

[ref46] Sabeena Farvin K. H. Baron C. P. Nielsen N. S. Otte J. Jacobsen C. (2010). Antioxidant activity of yoghurt peptides: part 2- characterisation of peptide fractions. Food Chem. 123, 1090–1097. doi: 10.1016/j.foodchem.2010.05.029

[ref47] Sahan N. Yasar K. Hayaloglu A. A. (2008). Physical, chemical and flavour quality of non-fat yogurt as affected by a β-glucan hydrocolloidal composite during storage. Food Hydrocoll. 22, 1291–1297. doi: 10.1016/j.foodhyd.2007.06.010

[ref48] Salih M. M. Hamid O. I. A. (2013). Effect of fortifying camel’s milk with skim milk powder on the physicochemical, microbiological and sensory characteristics of set yoghurt. Adv. J. Food Sci. Technol. 5, 765–770. doi: 10.19026/ajfst.5.3161

[ref49] Salminen S. Collado M. C. Endo A. Hill C. Lebeer S. Quigley E. M. M.. (2021). The international scientific association of probiotics and prebiotics (ISAPP) consensus statement on the definition and scope of postbiotics. Nat. Rev. Gastroenterol. Hepatol. 18, 649–667. doi: 10.1038/s41575-021-00440-6, PMID: 33948025 PMC8387231

[ref50] Sharafi H. Moradi M. Amiri S. (2022). Application of cheese whey containing postbiotics of *Lactobacillus acidophilus* LA5 and *Bifidobacterium animalis* BB12 as a preserving liquid in high-moisture mozzarella. Foods 11:3387. doi: 10.3390/foods11213387, PMID: 36359999 PMC9655881

[ref51] Sharma M. Shukla G. (2016). Metabiotics: one step ahead of probiotics; an insight into mechanisms involved in anticancerous effect in colorectal cancer. Front. Microbiol. 7:1940. doi: 10.3389/fmicb.2016.01940 27994577 PMC5133260

[ref52] Tamime A. Y. Barrantes E. Sword A. M. (1996). The effect of starch based fat substitutes on the microstructure of set-style yogurt made from reconstituted skimmed milk powder. Int. J. Dairy Technol. 49, 1–10. doi: 10.1111/j.1471-0307.1996.tb02612.x

[ref53] Thibault H. Aubert-Jacquin C. Goulet O. (2004). Effects of long-term consumption of a fermented infant formula (with *Bifidobacterium breve* c50 and *Streptococcus thermophilus* 065) on acute diarrhea in healthy infants. J. Pediatr. Gastroenterol. Nutr. 39, 147–152. doi: 10.1097/00005176-200408000-00004, PMID: 15269618

[ref54] Thorakkattu P. Khanashyam A. C. Shah K. Babu K. S. Mundanat A. S. Deliephan A.. (2022). Postbiotics: current trends in food and pharmaceutical industry. Foods 11:3094. doi: 10.3390/foods11193094 36230169 PMC9564201

[ref55] Wegh C. A. M. Geerlings S. Y. Knol J. Roeselers G. Belzer C. (2019). Postbiotics and their potential applications in early life nutrition and beyond. Int. J. Mol. Sci. 20:4673. doi: 10.3390/ijms20194673, PMID: 31547172 PMC6801921

[ref56] Yousefvand A. Huang X. Zarei M. Saris P. E. J. (2022). Lacticaseibacillus rhamnosus GG survival and quality parameters in kefir produced from kefir grains and natural kefir starter culture. Foods 11:523. doi: 10.3390/foods11040523, PMID: 35205998 PMC8871425

[ref57] Yu M. Ma J. Wang X. Lu M. Fu X. Zhang L.. (2022). Peanut sprout yogurt: increased antioxidant activity and nutritional content and sensory evaluation by fuzzy mathematics. J. Food Process. Preserv. 46:e16663. doi: 10.1111/jfpp.16663

[ref58] Zoidou E. Theodorou S. Moschopoulou E. Sakkas L. Theodorou G. Chatzigeorgiou A.. (2019). Set-style yoghurts made from goat milk bases fortified with whey protein concentrates. J. Dairy Res. 86, 361–367. doi: 10.1017/S0022029919000499, PMID: 31423963

